# Feasibility and safety of adenosine cardiovascular magnetic resonance in patients with MR conditional pacemaker systems at 1.5 Tesla

**DOI:** 10.1186/s12968-015-0218-x

**Published:** 2015-12-22

**Authors:** Oliver Klein-Wiele, Marietta Garmer, Rhyan Urbien, Martin Busch, Kaffer Kara, Serban Mateiescu, Dietrich Grönemeyer, Michael Schulte-Hermes, Marc Garbrecht, Birgit Hailer

**Affiliations:** Deptartment of Cardiology, Katholisches Klinikum Essen, University of Witten/Herdecke, Hülsmannstraße 17, 45355 Essen, Germany; Department of Radiology, Grönemeyer Institut Bochum, University of Witten/Herdecke, Universitätsstraße 142, 44799 Bochum, Germany; Cardiovascular Centre, Josef Hospital, University of Bochum, Gudrunstr. 56, 44791 Bochum, Germany; Department of Cardiology, Prosper-Hospital Recklinghausen, University of Witten/Herdecke, Mühlenstraße 27, 45659 Recklinghausen, Germany

**Keywords:** Pacemaker, MR conditional, Cardiovascular magnetic resonance, Adenosine stress, Safety, Sinus node dysfunction, Atrioventricular block, MR safe pacing modes, Asynchronous pacing

## Abstract

**Background:**

Cardiovascular Magnetic Resonance (CMR) with adenosine stress is a valuable diagnostic tool in coronary artery disease (CAD). However, despite the development of MR conditional pacemakers CMR is not yet established in clinical routine for pacemaker patients with known or suspected CAD. A possible reason is that adenosine stress perfusion for ischemia detection in CMR has not been studied in patients with cardiac conduction disease requiring pacemaker therapy. Other than under resting conditions it is unclear whether MR safe pacing modes (paused pacing or asynchronous mode) can be applied safely because the effect of adenosine on heart rate is not precisely known in this entity of patients. We investigate for the first time feasibility and safety of adenosine stress CMR in pacemaker patients in clinical routine and evaluate a pacing protocol that considers heart rate changes under adenosine.

**Methods:**

We retrospectively analyzed CMR scans of 24 consecutive patients with MR conditional pacemakers (mean age 72.1 ± 11.0 years) who underwent CMR in clinical routine for the evaluation of known or suspected CAD. MR protocol included cine imaging, adenosine stress perfusion and late gadolinium enhancement.

**Results:**

Pacemaker indications were sinus node dysfunction (*n* = 18) and second or third degree AV block (*n* = 6). Under a pacing protocol intended to avoid competitive pacing on the one hand and bradycardia due to AV block on the other no arrhythmia occurred. Pacemaker stimulation was paused to prevent competitive pacing in sinus node dysfunction with resting heart rate >45 bpm. Sympatho-excitatory effect of adenosine led to a significant acceleration of heart rate by 12.3 ± 8.3 bpm (*p* < 0.001), no bradycardia occurred. On the contrary in AV block heart rate remained constant; asynchronous pacing above resting heart rate did not interfere with intrinsic rhythm.

**Conclusion:**

Adenosine stress CMR appears to be feasible and safe in patients with MR conditional pacemakers. Heart rate response to adenosine has to be considered for the choice of pacing modes during CMR.

## Background

Cardiovascular magnetic resonance (CMR) as a non-invasive imaging modality is firmly established in the clinical workup for patients with known or suspected CAD. It has become the gold standard for chamber quantification and detection of left ventricular wall motion impairment in ischemic cardiomyopathy [1–3]. Post myocardial infarction (MI) complications such as negative remodeling, formation of aneurysms and intraventricular thrombi, pericardial effusion and ischemic mitral valve regurgitation can be detected precisely even in obese patients less suitable for echocardiographic assessment [4]. It has become the most important technique for tissue characterization such as scar detection in MI [5], and a stand alone imaging modality in differential diagnosis in ischemic versus other cardiomyopathies such as myocarditis [1]. Adenosine stress perfusion imaging plays a major role in the assessment of unknown coronary status [3, 6]. It has a class Ia level A recommendation in case of intermediate pre test probability of CAD in latest guidelines [3]. Hemodynamic relevance of stenoses in known CAD can be evaluated reliably [7].

In patients with sinus node dysfunction (SND) and AV-block underlying or concomitant CAD is common [8, 9]. In both disorders adenosine administration is only permitted with a permanent PM in situ due to possible bradycardia [10]. A high number of patients with SND or AV-block undergo PM implantation [11]; taken together both disorders constitute the majority of pacemaker indications worldwide [12].

In the past presence of a pacemaker was regarded as a contraindication for MR scanning [13, 14]. Nevertheless a number of studies have been conducted in these patients showing no relevant complications and sufficient overall image quality [15–19]. Recent development of MR conditional PM has opened this technology for patients with PM in clinical routine [20]. While safety of CMR without stress agents in patients with MR conditional PM has been shown in a number of studies [21–23], apart from single cases [17, 24] there is no data available on adenosine stress perfusion imaging in patients with PM neither conventional nor MR conditional.

In MR conditional PM only deactivation (ODO-mode) or asynchronous pacing (DOO, AOO, VOO) [25] are available to avoid inhibition by electromagnetic interference or tracking of electromagnetic signals. In SND and AV-block selecting an adequate pacing mode for routine adenosine stress CMR can be challenging because the effect of adenosine on heart rate (HR) is not precisely known in this entity of patients. On the one hand asynchronous mode (i.e. pacing at a fixed rate above baseline HR) could result in competitive pacing: HR can accelerate under adenosine [26] reaching the fixed pacing rate without inhibiting PM activity because sensing and inhibition is deactivated in this mode. PM stimulation could then fall in the vulnerable period of the cardiac cycle and trigger arrhythmia [27]. On the other hand deactivation of pacing in patients with normal HR under resting conditions could result in bradycardia or asystole under adenosine [10].

In conclusion the high value of adenosine stress CMR in known or suspected CAD is well established but it is still unknown whether the method is safe and feasible in PM patients with SND or AV block who are programmed to the restricted pacing modes required by MRI conditional devices during performance of CMR. We investigate for the first time feasibility and safety of CMR in clinical routine in PM patients.

## Methods

We retrospectively analyzed MR scans of 24 consecutive patients with MR conditional PM who underwent routine adenosine stress CMR for the evaluation of known or suspected CAD including cine imaging, adenosine stress perfusion and late gadolinium enhancement after informed consent was obtained from March 2014 to April 2015. The study complied with the Declaration of Helsinki and was approved by the local Institutional Review Board (University of Witten/Herdecke, Medical Faculty).

### Pacemaker programming

CMR was performed more than six weeks after PM implantation in all individuals according to ESC guidelines [13]. Prior to CMR imaging battery status of the device, lead impedance, pacing capture thresholds and sensing amplitudes were measured.

Devices were set to MR safe mode according to manufacturer’s instructions immediately prior to the scan and reprogrammed immediately thereafter. Programming was performed according to a predefined protocol: To avoid interference of intrinsic rhythm with PM-stimulation in patients with SND and resting heart rate HR > 45 bpm no pacing (ODO)-mode was engaged during the scan - also when atrial fibrillation (AF) was present at the time of the scan. In individuals with SND and HR ≤ 45 bpm the pacemaker was set to asynchronous atrial stimulation (AOO, 60 bpm). All patients with intermittent or permanent second or third degree AV block were continuously paced in asynchronous mode irrespective of their actual rhythm and HR to avoid possible asystole or bradycardia due to worsening AV conduction induced by adenosine. Pacing rate was set 10 bpm above spontaneous heart rate with a minimum of 60 bpm. VOO mode at 60 bpm was chosen in AV block with sinus rate > 45 bpm to avoid competitive atrial stimulation, DOO mode at 60 bpm in AV block with sinus bradycardia ≤ 45 bpm. Patients in AF at the time of the scan were paced VOO at 60 bpm if resting heart rate was ≤ 45 bpm. Table 1 shows the pacing protocol used to select pacing modes for specific clinical constellations.

**Table 1:** Protocol for the selection of pacing modes

	Sinus rate > 45 bpm	Sinus rate ≤ 45 bpm
Sinus node dysfunction without AV-block > I°	ODO	AOO at 60 bpm
AV-block > I° (present or history of)	VOO at 10 bpm > IHR	DOO at 60 bpm
Atrial fibrillation at time of scan		VOO at 60 bpm when HR <45 bpm

*bpm*, Beats per minute, *AV* Atrioventricular, *IHR* Intrinsic heart rate

### Safety precautions

Patients were monitored during the scan with continuous electrocardiographic and visual supervision by a cardiologist present in the scanner room. Voice contact was maintained with the patient at all times of the scan. Advanced cardiac life support protocol was in effect. In the scanner the patient was placed on a carry sheet; medical staff was trained for rapid removal of the patient from the scanner room in the event of cardiopulmonary compromise. Thus immediate treatment of severe arrhythmia and reactivation of PM stimulation within seconds in non-paced patients was guaranteed. Atropine, adrenaline and theophylline injections were prepared ready for use in case of bradycardia. Two separate cubital venous cannulas were used for adenosine and gadolinium contrast agent respectively.

### Cardiovascular magnetic resonance

CMR was performed with a 1.5 T wide bore system (ESPREE – Siemens Healthcare, Erlangen, Germany) using a 4-channel body array and an 8-channel spine coil. Maximum gradient field was 33 mT/m (Z-Engine) with a slew rate of 100 T/m/s. Maximum specific absorption rates were limited to 2.0 W/kg.

Our standard protocol meets the Society of Cardiovascular Magnetic Resonance (SCMR) standards for CMR [1]. Cine steady-state free precession (SSFP) gradient-echo images were obtained in 10 to 12 short axis slices depending on the size of the ventricles and in 3 long axis planes corresponding to two, three and four chamber views. For stress perfusion-imaging adenosine was administered as 3-min infusion of 140ug/kg body weight/min. First-pass perfusion imaging was carried out with intravenous bolus administration of gadolinium (0.2 mmol/kg body weight) in a fast low angle shot (FLASH) sequence (3 to 4 slices). Late Gadolinium Enhancement (LGE) images were acquired fifteen minutes after injection of gadolinium as phase-sensitive inversion-recovery (PSIR) in short (10 to 12 slices) and long axis (3 planes) views. Table 2 shows details of the MR protocol.

**Table 2:** Details of the CMR protocol

Objective	Sequence	Plane	TR/TE (ms)	Slice thickness (mm)	Slices	Matrix	FOV Phase (mm)	Flip angle	PAT
Anatomical orientation	HASTE	Axial, coronal, sagittal	1000/44	8	27/35	125×256/142×256	290/360	160	1
Cine imaging	True FISP	Long and short axes	66/1.6	8	LA: 3 SA: 10/12	166×256	300/370	79	1
First pass perfusion	GRE	Short axes	176/1.2	8	3/4	96×128	260/300	15	1
Late Gadolinium Enhancement	PSIR	Long and short axes	1024/3.5	8	LA: 3 SA: 10/12	144×256	270	25	0

*HASTE* Half fourier acquisition single shot turbo spin echo, *LA* Long axis, *SA* Short axis, *TR* Repetition time, *TE* Echo time, *FOV* Field of view, *PAT* Parallel acquisition technique, True *FISP*, True fast imaging with steady state precession, *GRE* Gradient echo, *PSIR* Phase-sensitive inversion recovery

## Results

### General characteristics

Twenty-four CMR examinations were analyzed. Patients had a mean age of 72.1 ± 11.0 years. 11 (45.8 %) had known CAD, 7 (29.1 %) previous MI. All other patients had intermediate pretest probability of CAD [28]. Echocardiography had shown preserved systolic left ventricular (LV) function in all subjects. Pacemaker indications were sinus node dysfunction (SND) (*n* = 18; 75 %) and second or third degree AV-block (*n* = 6; 25 %). No patient was PM dependent (HR <30 bpm). Impulse generator/lead models were Advisa (*n* = 5; 20.8 %) and Ensura (*n* = 18; 75 %) MRI SureScan/CapSureFix 5076 Novus (atrial), CapSureSense 4074 (ventricular) (Medtronic Inc., Minneapolis, MA, USA); Entovis DR-T/Safio S 53 (atrial), Safio S60 (ventricular) (Biotronik SE & Co. KG, Berlin, Germany), *n* = 1, 4.2 %. For detailed baseline characteristics see Table 3.

**Table 3:** Baseline characteristics

Total patients	24	
Mean Age (years)	72.1 ± 11.0	
	N	%
Female	5	20.8
Pacemaker indication		
ᅟHigher degree AV Block	6	25.0
ᅟSinus node dysfunction	18	75.0
Coronary artery disease	11	45.8
Paroxysmal atrial fibrillation	10	41.7
Hypertension	19	79.2
Impaired renal function	4	16.7
Previous Stroke	7	29.2
Pacemaker		
ᅟEnsura DR MRI Sure Scan EN1DR01	18	75.0
ᅟAdvisa DR MRI Sure Scan A3DR01	5	20.8
ᅟEntovis DR-T	1	4.2
Implantation site left pectorally	16	66.7
Pacemaker dependent	0	
Pacing mode during Scan		
ᅟODO	17	70.8
ᅟAOO	1	4.2
ᅟVOO	5	20.8
ᅟDOO	1	4.2

*AV* Atrioventricular

### Effect and safety of adenosine administration for stress perfusion

There were no adenosine induced adverse events.

In 17 patients with SND and normal AV-conduction (*n* = 14) or normofrequent AF (*n* = 3) at the time of the scan the pacemaker stimulation was deactivated (ODO). Adenosine administration accelerated mean HR by 12.3 ± 8.3 bpm (*p* = 0.001). AV-conduction was not significantly influenced by adenosine; no higher degree AV block occurred. When sinus rate was <45 bpm (*n* = 1) AOO pacing at 60 bpm led to permanent capture, no acceleration of HR under adenosine was noticed.

In patients with second or third degree AV block and sinus rate >45 bpm (*n* = 5) that were paced asynchronously no arrhythmia was detected; permanent ventricular capture was seen on ECG monitoring during the scan. One patient with intermittent second degree AV block but with normal AV conduction at the time of CMR was paced 10 bpm above spontaneous HR. When sinus rate was <45 bpm (*n* = 1) D00 pacing at 60 bpm was engaged and did not lead to competitive atrial stimulation. No competitive ventricular stimulation was observed. Adenosine did not induce tachycardia.

**Fig. 1:**
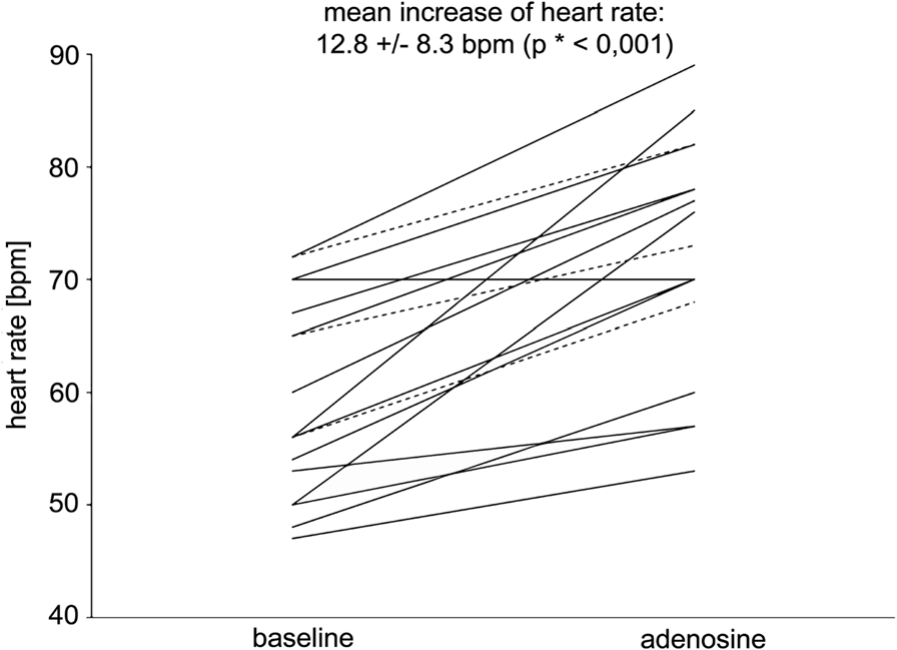
Adenosine effect on heart rate in sinus node dysfunction. Individual changes of heart rate in 17 non-paced patients with SND in SR or momentary AF under adenosine administration, solid lines: sinus rhythm, dotted lines: AF, *paired *t*-test. SND, sinus node dysfunction; SR, sinus rhythm, AF, atrial fibrillation

Figure 1 summarizes individual HR response under adenosine in non-paced patients with SND.

### Device integrity

Device integrity was not compromised by the CMR scan. Lead impedance was unchanged pre and post CMR for atrial and ventricular leads. Pacing capture thresholds were equally unaffected. Sensing amplitudes remained unchanged as well as battery voltage. Table 4 summarizes device parameters pre and post CMR.

**Table 4:** Comparison of device parameters before and after CMR

	Before MR	After MR	P*
P-wave amplitude (mV)	2.87 ± 1.86	3.10 ± 1.70	0.32
R-wave amplitude (mV)	12.27 ± 5.32	12.05 ± 5.44	0.59
Atrial lead impedance (Ohm)	469 ± 61	468 ± 65	0.65
Ventricular lead impedance (Ohm)	601 ± 120	603 ± 118	0.57
Atrial PCT (V@0.4 ms)	0.66 ± 0.25	0.66 ± 0.20	1.0
Ventricular PCT (V@0.4 ms)	0.63 ± 0.26	0.55 ± 0.28	0.1
Battery voltage (V)	2.97 ± 0.42	2.97 ± 0.42	n.a.

*CMR* Cardiovascular magnetic resonance, *PCT* Pacing capture threshold, *Wilcoxon signed rank test

### Diagnostic value of cine sequences, late gadolinium enhancement and adenosine stress perfusion

CMR showed preserved ejection fraction in all patients. Image quality was sufficient to calculate ejection fraction in long axis views despite moderate PM artifacts in all patients and corresponded to echocardiographic findings. Regional wall motion impairment was seen in 6 (25.0 %), LV hypertrophy in 7 (29.2 %) patients. Minor valve dysfunction was found in 3 (12.5 %) patients, aortic aneurysm > 45 mm in 2 (8.3 %) patients. LGE with subendocardial or transmural distribution pattern corresponding to post MI scarring was present in 7 (29.2 %) patients and was not obscured by PM artifacts caused by generator or leads. Postinflammatory myocardial scarring was seen in one patient. Adenosine induced perfusion deficit was visible in two patients. One patient consecutively underwent percutaneous coronary intervention with stent implantation in the right coronary artery; the second patient was scheduled for bypass surgery.

**Fig. 2:**
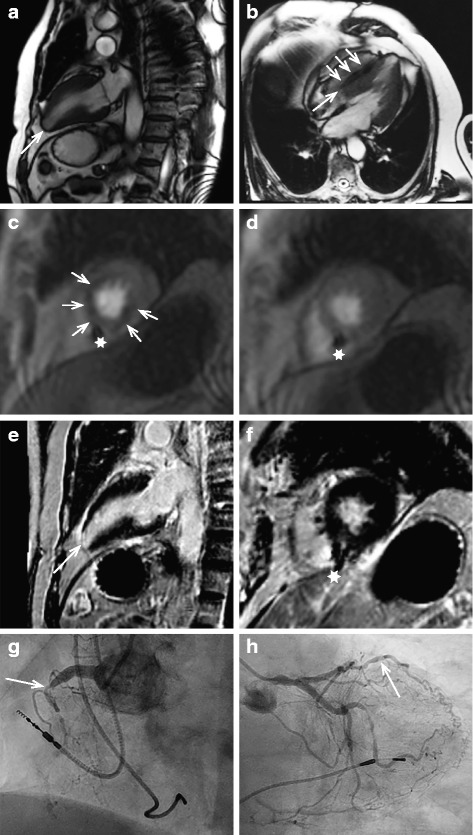
Adenosine stress CMR and subsequent coronary angiogram in a patient with AV block, suspected coronary artery disease and pathologic CMR. Cine imaging (**a**, **b**) shows small apical aneurysm (a, arrow); myocardium can be delineated (b, arrow) despite PM lead artifact (small arrows). Stress perfusion shows perfusion deficit in LAD and RCA territory (c, small arrows) not visible in resting perfusion (**d**); PM lead artifact is visible (asterixes). LGE (**e**/**f**) shows myocardial scarring apically (arrow) and viable myocardium in the ischemic area seen on stress perfusion (**c**). No major compromise of image quality by PM artifacts is present. Coronary angiogram corresponds to CMR findings with chronic total occlusion of RCA (**g**, arrow) and LAD (**h**, arrow). CMR, Cardiovascular Magnetic Resonance; AV, atrioventricular; PM, pacemaker; LAD, left anterior descendent coronary artery; RCA, right coronary artery; LGE, Late Gadolinium Enhancement

See Fig. 2 for examples of cine sequences, first pass perfusion and LGE in a patient without previously known CAD. Stress perfusion compared to resting perfusion shows a perfusion deficit in viable myocardium corresponding to consecutive invasive coronary angiogram.

## Discussion

The present study shows no complications of adenosine stress CMR related to the presence of a PM or the underlying cardiac conduction disorder. The device remained intact; no arrhythmia was induced by adenosine in this highly selected entity of patients.

### Safety of adenosine administration

Apart from influence of the MR field on device function, the most important safety issue in adenosine stress CMR is the MR conditional pacing mode itself. Only asynchronous pacing without sensing or deactivation can be programmed in this mode to avoid tracking of electromagnetic impulses and inhibition by electromagnetic interference [13]. Thus two possible risks should be addressed: proarryhythmia due to competitive pacing in the vulnerable period of the cardiac cycle on the one hand, bradycardia or asystole due to missing backup pacing on the other. Some investigators estimate the risk of asynchronous pacing to be low [29]. However, for routine adenosine stress CMR in CAD sequences for localization, cine imaging, first pass perfusion and LGE are necessary - with additional sequences (tissue characterization, flow analysis) even longer periods in MR conditional mode may be required. Paused or asynchronous PM stimulation may be of relevance under these conditions.

This issue is complicated by the effect of adenosine on HR during stress perfusion: The negative dromotropic effect of the substance on the cardiac conduction system may result in bradycardia or asystole in patients with paused PM stimulation [30, 31]. On the other hand direct receptor-specific stimulation of sympathetic afferences can result in sinus tachycardia that can interfere with fixed pacing rates in asynchronously paced individuals [32, 33].

The present data suggests that in SND with normal resting HR and preserved AV conduction paused PM stimulation (ODO mode) is suitable for adenosine stress perfusion. Apart from one patient with constant HR we found a significant increase in HR i.e. no negative dromotropic effect of adenosine in SND with an adenosine dosage of 140ug/kg/min limited to three minutes. Thus the direct sympatho-excitatory effect of adenosine overrides cardiac inhibition comparable to patients without SND. In AF we also found predominance of the sympatho-excitatory reflex with positive chronotropy. Choosing asynchronous pacing would have been problematic in SND because adenosine accelerated HR by up to 29 bpm. Pacing far above baseline HR for a longer time could cause discomfort or even circulatory compromise in PM patients adapted to relative bradycardia [34].

We propose asynchronous pacing in AV block because the risk of asystole under adenosine is high [35], persistent AV-block after cessation of adenosine infusion has been described [36]. HR remained constant because AV conduction was impaired and increase in sinus rate due to sympathetic stimulation did not translate into tachycardia making competitive pacing unlikely. However this may depend on the severity of the AV conduction disorder. Functional conduction delay may be overcome; structurally damaged AV conduction in higher degree AV block is unlikely to recover under sympathetic stimulation. In patients with intermittent AV block but normal AV conduction pre CMR we chose pacing only 10 bpm above resting HR, however this may not always be adequate. AV conduction could stay intact even under adenosine allowing acceleration of HR beyond the fixed pacing rate. The optimum pacing rate in this group of patients has to be evaluated in larger studies; even inactivation of the pacemaker could be adequate in AV block with normal AV conduction at the time of MR provided that immediate cessation of adenosine infusion and fast reactivation of pacing are guaranteed.

In the present study no proarrhythmia was observed under individually adapted pacing modes; nevertheless arrhythmia is the main safety issue. Calculating the risk of arrhythmia one has to take into account that severe brady- or tachycardia has been described almost exclusively for bolus administration of adenosine; malignant reentrant tachycardia due to accessory pathways is predominant [37]. In continuous slow application of adenosine persistent and life threatening bradycardia is unlikely to occur due to the short half-life of only several seconds [35]; on the contrary severe arrhythmia induced by competitive pacing may persist [27, 38]. Nevertheless caution in this warranted. Pharmacologic therapy of arrhythmia and reactivation of paused PM stimulation must be available immediately. For ischemia detection the possible risk of arrhythmia in adenosine stress CMR under asynchronous or deactivated pacing should be weighed against possible risks and diagnostic limitations of other non-invasive tests like stress echo and scintigraphy or invasive coronary angiography. Thus the value of CMR in the workup of CAD in PM patients has to be compared to other diagnostic strategies, namely when the high supervisory expense in this setting is considered. We encourage prospective randomized studies to clarify which imaging strategy is the best choice for PM patients in term of safety and clinical value.

### Diagnostic value

While several publications have noted rather minimal artifact and the ability to produce diagnostic scans, others have noted compromised CMR images because of artifact [39]. In this study PM artifacts caused no clinically relevant compromise of image quality. In AV block the principle of ischemia detection by adenosine should be unaffected by lack of heart rate response because relative ischemia is induced by vasodilatation via cardiac A1 receptors and not by positive chronotropy like in dobutamine stress [40]. However increase in heart rate as a marker of adenosine response is unavailable in those patients; side effects of adenosine like respiratory symptoms may be no reliable indicator for a systemic effect of adenosine. The splenic switch-off sign as described by Mainsty et al. [41] may be a helpful indicator to detect insufficient adenosine stress requiring higher adenosine dosage. Adenosine stress perfusion for ischemia detection has been studied in single photon emission computed tomography imaging and scintigraphy [42] but not in CMR. As proof of concept in this study severe CAD could be detected in one patient with AV block and perfusion deficit under adenosine. Larger prospective studies have to confirm diagnostic value of stress perfusion MR in this subgroup of patients.

### Device integrity

The present data on lead integrity are in line with previous studies on MR conditional PM [22, 23] showing no clinically significant alterations of lead impedance, pacing capture threshold and sensing amplitude. Significantly reduced battery voltage (BV) immediately after MR has been described for MR conditional models [21]. Thus in theory repeated MR scans could result in reduced longevity of the systems. We found unchanged battery status post CMR in all patients. Thus our results support the finding of Claas et al. [43] showing no decrease of BV above the accuracy of measurement post MR. Clinically relevant reduction of BV by routine adenosine stress CMR in patients with MR conditional PM is unlikely taking also into account that a decrease of 0.05 V does not seem to reduce longevity of the PM to a clinically relevant extent [44].

### Limitations

This study is limited by the small sample size and the lack of a control group. Adverse effects may only appear in a larger cohort of patients. No intermediate or long-term follow up data was provided. Moreover the diagnostic value of CMR was not evaluated invasively in patients without perfusion deficit.

## Conclusion

Our data suggest adenosine stress CMR in patients with MR conditional PM to be feasible and safe for the workup of CAD. We propose individualized pacing modes to reduce the risk of proarrhythmia that have to be further evaluated. Adenosine induced sympathetic stimulation overrides inhibitory effects on the conduction system leading to positive chronotropy only in patients with intact AV conduction but not in higher degree AV block. We encourage further research to determine the diagnostic value of adenosine stress CMR in PM patients and to establish guidelines on pacemaker programming for adenosine stress in clinical routine.

## Abbreviations

AV: Atrioventricular
BV: Battery voltage
CAD: Coronary artery disease
CMR: Cardiovascular magnetic resonance
ESC: European society of cardiology
FLASH: Fast low angle shot
GRE: Gradient echo
HASTE: Half fourier acquisition single shot turbo spin echo
HR: Heart rate
LAD: Left anterior descendent coronary artery
LGE: Late gadolinium enhancement
LV: Left ventricular
MI: Myocardial infarction
MR: Magnetic resonance
PCT: Pacing capture threshold
PM: Pacemaker
PSIR: Phase-sensitive inversion recovery
RCA: Right coronary artery
SCMR: Society for cardiovascular magnetic resonance
SND: Sinus node dysfunction
SSFP: Steady-state free precession
STIR: Short-tau inversion recovery
True FISP: True fast imaging with steady state precession
T1wTSE: T1 weighed turbo spin echo
VencGRE: Velocity encoding gradient echo
